# Short-term and long-term solar irradiance forecasting with advanced machine learning techniques in Zafarana, Egypt

**DOI:** 10.1038/s41598-025-24853-4

**Published:** 2025-11-12

**Authors:** Ali Taha, Peter Makeen, Nathalie Nazih

**Affiliations:** 1 Electrical Engineering Department, Faculty of Engineering, The British University in Egypt, El-Sherouk City, Egypt; 2 Department of Electronic & Electrical Engineering, University of Bath, Bath, UK

**Keywords:** Solar energy, Wind energy

## Abstract

The increasing demand for renewable energy sources has positioned solar energy as a pivotal component in the global transition towards sustainable power generation. As the demand increases for solar energy production, the need for technical specifications, resource cost increases, and output power prediction increases. Thus, recent studies in machine learning (ML) and deep learning (DL) techniques have opened new ways for improving solar irradiance predictions by leveraging historical data. This paper proposes an integrated framework for forecasting solar irradiance, combining feature selection techniques with machine learning models to address region-specific challenges in Zafarana, Egypt, aimed at improving predictive accuracy using historical data sourced from the NASA Power Project for both short-term and long-term horizons. The framework begins with feature selection techniques, including One-Way ANOVA, Boruta, and Random Forest, to identify key variables influencing solar irradiance. This is followed by the implementation of ML and DL models, including Linear Regression (LR), Decision Tree (DT), Gradient Boosting (GB), Random Forest (RF), Convolutional Neural Network (CNN), Long Short-Term Memory (LSTM), and a hybrid CNN-LSTM model. The analysis reveals that RF and GB achieved high accuracy, with R² scores of 0.9948 and 0.9724, respectively, for one-day forecasts and 0.978 and 0.954, respectively, for one-month forecasts. The results indicate that the proposed machine learning approaches significantly outperform traditional forecasting methods, demonstrating their potential for optimizing solar energy management.

## Introduction

The uprising interest in renewable energies increases the market growth to produce energy resources like solar and wind^1^. Solar energy is the most preferable power source in sustainability and usage worldwide and an alternative source to fossil fuel^2^. However, solar energy production is related to the resource’s costs and meteorological stationery needs, like the demands for the photovoltaic cell’s materials with variable technical specifications. However, power prediction is regarded as a key challenge for PV systems. Machine learning predictive models are introduced to forecast PV output power using historical time series data^3^. Several studies benefited from the use of machine learning algorithms for solar irradiance forecasting, like the deep learning method that showed promising results. Hybrid models combining machine learning with clustering have offered alternative solutions for location-specific predictions. Ribeiro et al.^4^ developed a clustering-based framework to analyze solar irradiance and wind dynamics across various regions in Brazil, applying K-Means, K-Medoids, and SOM algorithms. The K-Means algorithm, for example, achieved silhouette coefficients of 0.63 and 0.46 on solar data from Floresta and Janaúba, highlighting clustering’s effectiveness in capturing spatial patterns. In parallel, studies like those by Zhang et al.^1^ incorporated data fusion and error correction models. Zhang’s approach integrated meteorological data, delayed power information, and sensor data into a Dynamic Bayesian Network (DBN) to improve forecasting accuracy over traditional models such as Support Vector Regressor (SVR) and K-Nearest Neighbor (KNN). This model achieved a near-optimal R² of 0.98, outperforming others like Artificial Neural Network (ANN) and Long Short-Term Memory (LSTM), underscoring the benefits of fusing different data types for error mitigation. Advanced machine learning models for solar irradiance forecasting were introduced by^5^, focusing on the Rectified Linear Unit Activation with Adaptive Moment Estimation Neural Network (RELAD-ANN) and Linear Support Vector Machine with Individual Parameter Features (LSIPF) architectures to address computational efficiency and predictive accuracy. The RELAD-ANN model achieved an R² score of 0.935, MAE of 8.20, and MAPE of 3.48%, outperforming traditional models like LSTM and 1D-CNN, while the LSIPF model exhibited limited predictive capability. Light GBM further demonstrated robustness in evaluating environmental influences on solar irradiance, surpassing Support Vector Regression (SVR) in accuracy.

Asynchronous dual-pipeline framework for real-time solar irradiance forecasting using deep learning models was proposed in^3^. The framework consists of a training process that continuously updates the model with recent instances of data, and as soon as new input data is available, the prediction process generates forecasts based on the updated model. The models used in the process include Multilayer Perceptron (MLP), LSTM, Convolution Neural Network (CNN), and Transformers, with data from a Canadian PV solar plant. Their findings indicated that MLP yielded the lowest mean absolute error (MAE) at 8.034, while transformer-based models exhibited the highest error and variability. Similarly, Hari et al.^6^ focused on LSTM architecture, finding that stacked LSTM layers improved performance significantly, reducing root mean square error (RMSE) from 682.4 for single-layer LSTM to as low as 83.86 for three-layer LSTM, demonstrating the advantage of model depth in prediction accuracy. A short-term solar irradiation forecasting with a multilayer artificial neural network trained on data from Gandon in Senegal^7^, achieving high accuracy with an R² score of 0.984 for a 30-minute horizon. This performance was comparable to other neural network-based approaches, including those demonstrated by Jebli et al.^2^, where the ANN achieved an R² of 0.93, outperforming traditional models like linear regression and support vector regression. Soni et al.^8^ focused on the improvement of MLP neural network algorithm accuracy with mean square error as a performance metric, achieving 0.0226. Estimating the ultraviolet part of the solar spectrum using an artificial neural network used with global horizontal irradiation and the solar zenithal angle was focused by Fatima et al.^9^. Two conditions were compared: the clear days condition with RMSE 1.6% to 3% and $\:{R}^{2}$ score 0.99 and the nonclear days condition with RMSE 4% to 9% and $\:{R}^{2}$ score 0.96. Forecasting solar irradiance using hybrid machine learning models was explored by^10^. The study introduced two novel approaches: the Sequential Deep Artificial Neural Network (SDANN) for capturing complex weather patterns, and the Deep Hybrid Random Forest Gradient Boosting (RFGB) model, which outperformed benchmarks like XGBOOST and GRU. RFGB achieved superior accuracy with an MSE of 147.22, MAE of 8.77, and R² of 0.80, demonstrating its effectiveness in balancing computational efficiency and prediction reliability. Ablation studies further validated the impact of meteorological features on model performance, underscoring the method’s adaptability for renewable energy applications. A hybrid forecasting model for solar global horizontal irradiance using Bidirectional Long Short-Term Memory (BiLSTM) with iterative filtering (IF) was proposed by^11^. The model decomposed historical time series data into intrinsic mode functions via IF and employed partial autocorrelation for feature selection, alongside grid search for hyperparameter tuning. Compared to standalone models (LSTM, GRU, BiLSTM) and CEEMDAN-BiLSTM, the proposed approach achieved superior performance for 15-minute-ahead forecasts in Gangtok, India, with RMSE (6.873–11.775 W/m²), MAE (3.376–6.192 W/m²), and correlation coefficients (0.996–0.998). Additional validation included multi-horizon forecasts (15–60 min) and analysis across seasons and day types. Forecasting global horizontal irradiance (GHI) using LSTM with adaptive training algorithms was investigated by^12^. The study compared three training algorithms—ADAM, SGDM, and RMSprop—for a one-step-ahead LSTM model, tested on monthly data. Results demonstrated that ADAM outperformed other algorithms, achieving an annual average RMSE of 68.62 W/m² and MAPE of 10.30%, highlighting its efficacy for solar irradiance prediction. Forecasting solar irradiance using hybrid deep learning models was investigated by Kumar et al.^13^ for hour-ahead predictions in Indian locations. Eleven models, including Feedforward neural network (FFNN), LSTM, and wavelet-based hybrids (WPD), were evaluated. The WPD model achieved the lowest errors, with annual RMSE of 10.49–11.35 W/m² and MAE of 6.04–6.41 W/m², while maintaining high reliability (PICP 80.66–81.27% at 99% confidence). Hyperparameters were optimized via grid search, and input lags were selected using ACF/PACF analysis.

A sliding window approach combined with first-order differencing for very short-term solar irradiance forecasting using deep learning models proposed in^14^. Their approach utilized a CNN-LSTM architecture and achieved impressive results with an R² score of 0.979, RMSE of 34.89, and MAE of 20.07 for a prediction period of 1 h and 30 min. The study highlighted the effectiveness of deep learning models in handling time-series data for solar irradiance forecasting, particularly when incorporating meteorological features such as temperature and relative humidity. In^15^ a hybrid approach combining arithmetic optimization with deep learning for solar radiation prediction. Their model, which integrated the Arithmetic Optimization Algorithm (AOA) with CNN-LSTM, achieved remarkable results with an R² score of 100, MAE of 0.32, and RMSE of 0.43. Although specific details about the dataset and prediction period were not provided, the study underscored the potential of hybrid optimization techniques in enhancing the performance of deep learning models for solar radiation forecasting.

In^16^ a fuzzy inference system based on multi-type features fusion for intra-hour solar irradiance forecasts was introduced. Their approach involved fuzzing ground-based cloud images and numerical time series data, followed by the development of hierarchical fuzzy inference systems (HFIS) and an adaptive neuro-fuzzy inference system (ANFIS) optimized with particle swarm optimization (PSO). The model achieved an R² score of 0.936, MAE of 0.16, and RMSE of 0.29 for a 10-minute prediction period. This study demonstrated the potential of hybrid fuzzy systems in improving the accuracy of short-term solar irradiance predictions was focused in^17^. Intra-day global horizontal irradiance forecasting using the FY-4 A clear sky index and the Heliosat-2 model. Their full physical model aimed to simulate the transfer process of solar radiation in the Earth’s atmosphere system. The study reported an MAE of 75.07 and RMSE of 123.92 for a 180-minute prediction period. While the results indicated room for improvement, the use of satellite data and physical models provided a robust framework for solar irradiance forecasting.

Several works focused on empirical and physics-based methods for solar prediction. For instance, Teyabeen et al.^18^ evaluated seven empirical models on monthly forecasting, finding that the Sunshine quadratic model yielded the best performance on Libyan solar data, with an RMSE of 0.0195 and R² of 0.929. Similarly, Arif et al.^19^ used the ASHRAE model for clear-sky conditions on hourly predictions based on self-collected data, achieving varying RMSE values depending on seasonal conditions. awab et al.^20^, for instance, reduced Mean Absolute Percentage Error (MAPE) to 6.64 in Multan, 8.97 in Islamabad, and 14.14 in Peshawar with an ANN trained on NASA Power data. It was concluded that region-specific models using ANN have proven effective for real-time and daily predictions. In Manokwari, Beni et al.^21^ proposed a model built with the ELM algorithm for daily solar irradiation prediction using the NASA project for data, resulting in performance with a mean absolute error of 0.6392. In Algeria, Halima et al.^22^ employed ANFIS to predict daily global solar radiation, achieving high R² scores of 0.92–0.93 across different regions, emphasizing the suitability of adaptive models for diverse climatic conditions.

Advancements in transformer models for solar forecasting were comprehensively reviewed by^23^, emphasizing their role in grid integration and renewable energy systems. The study evaluated single, hybrid, and specialized transformer architectures across short- to medium-term forecasting horizons, demonstrating improved accuracy and computational efficiency. Key findings highlighted the models’ ability to handle complex solar data, with performance heavily influenced by hyperparameter tuning. Challenges such as high computational demands and dataset requirements were noted, alongside recommendations for standardizing configurations and advancing long-term forecasting. A transformer-based hybrid model for solar irradiance forecasting was proposed by^24^. The study introduced a Transformer-Infused Recurrent Neural Network (TIR) combining BiLSTM and GRU architecture with attention mechanisms and positional encoding. The TIR model achieved superior performance with an R² score of 0.9983, RMSE of 0.0140, and MAE of 0.0092, outperforming standalone ANN, BiLSTM, GRU, and Transformer models in handling meteorological data complexity and outliers.

Offloading and scheduling healthcare workflows in IoMT fog-cloud networks using deep reinforcement learning and blockchain was investigated by^25^. The study formulated the problem as a Markov decision process and proposed a multi-criteria offloading system based on deep reinforcement learning policies, combined with blockchain task scheduling. Simulation results demonstrated reduced communication and computation times for healthcare applications in dynamic IoMT environments. Accurate vehicle logo recognition using a category-consistent deep network was proposed by^26^. A convolutional-neural-network-based feature extraction model was developed to integrate high- and low-level image features, alongside a category-consistent mask learning module to eliminate reliance on license plate detection or manual annotations. The framework achieved superior performance on benchmark datasets (HFUT, XMU, CompCars, and VLD-45) by optimizing classification and category-consistency losses iteratively. A deep learning-based diagnostic framework for engine defect identification in two-wheeler vehicles was proposed by^27^. Vibration data were transformed into angular domain signals and processed using wavelet synchro-squeezed transform (WSST) to generate time-frequency images. By introducing an entropy-based regularization function to the CNN cost function, the method achieved a 3.8% higher accuracy compared to existing diagnostic techniques, demonstrating superior performance in detecting internal combustion engine defects.

Based on the literature review, various articles proposed the concept of solar irradiance prediction based on short-term and long-term forecast horizons using different machine learning models at different areas with specific parameters as summarized in Table 1. This table reveals the novelty of our paper compared with the current literature.

**Table 1:** Comparison between proposed models across various Studies.

Reference	Model	Time Period	Location	*R*²	MAE	RMSE	MSE
Halima et al.^22^	ANFIS	Daily	Ouargla city, Algeria	0.9304	NA	NA	NA
Halima et al.^22﻿^	GA-ANN	Daily	Ouargla city, Algeria	0.9076	NA	NA	NA
Jebli et al.^2^	MLP	Daily	Pirapora, Brazil	0.95	NA	NA	NA
Zhang et al.^1^	DBN	Daily	UK	0.98	41.95	109.82	NA
Nkounga et al.^7^	ANN	6 h	North-West Sengal	0.961	NA	0.075	NA
Rehiara et al.^21^	ELM	Daily	Manokwari, Papua Province	NA	0.6392	NA	NA
Nawab et al.^20^	ANN	Daily	Multan	NA	NA	0.35	NA
This Paper	Decision Tree	Daily	Zafarana, Egypt	0.9918	20.42	46.25	2139.4
	Gradient Boosting			0.9724	33.92	50.61	2562.1
	Random Forest			0.9948	15.77	34.72	1205.63

This paper presents a novel approach for predicting solar irradiance with minimal losses in comparison to existing literature. The approach comprises three key stages: first, unlike existing studies relying on one feature selection, this study evaluates three feature selection techniques; second, model implementation, showcasing machine learning algorithms outperform deep learning models in both short-term and long-term forecasts in this region; and third, solar irradiance forecasting. This paper is organized as follows: Sect. 2 gives an overview of the proposed approach, starting with the obtained data with the periodic time used and the required features. In addition to demonstrating three feature selection methods that highlight the best features obtained from the data and discussing the methodology of the solar irradiance prediction models and algorithms with the specification of the hardware and software used. Section 3 discusses the results obtained from the tested models with evaluation and discussion of different metrics.

## Data and methods

### The proposed approach

**Fig. 1:**
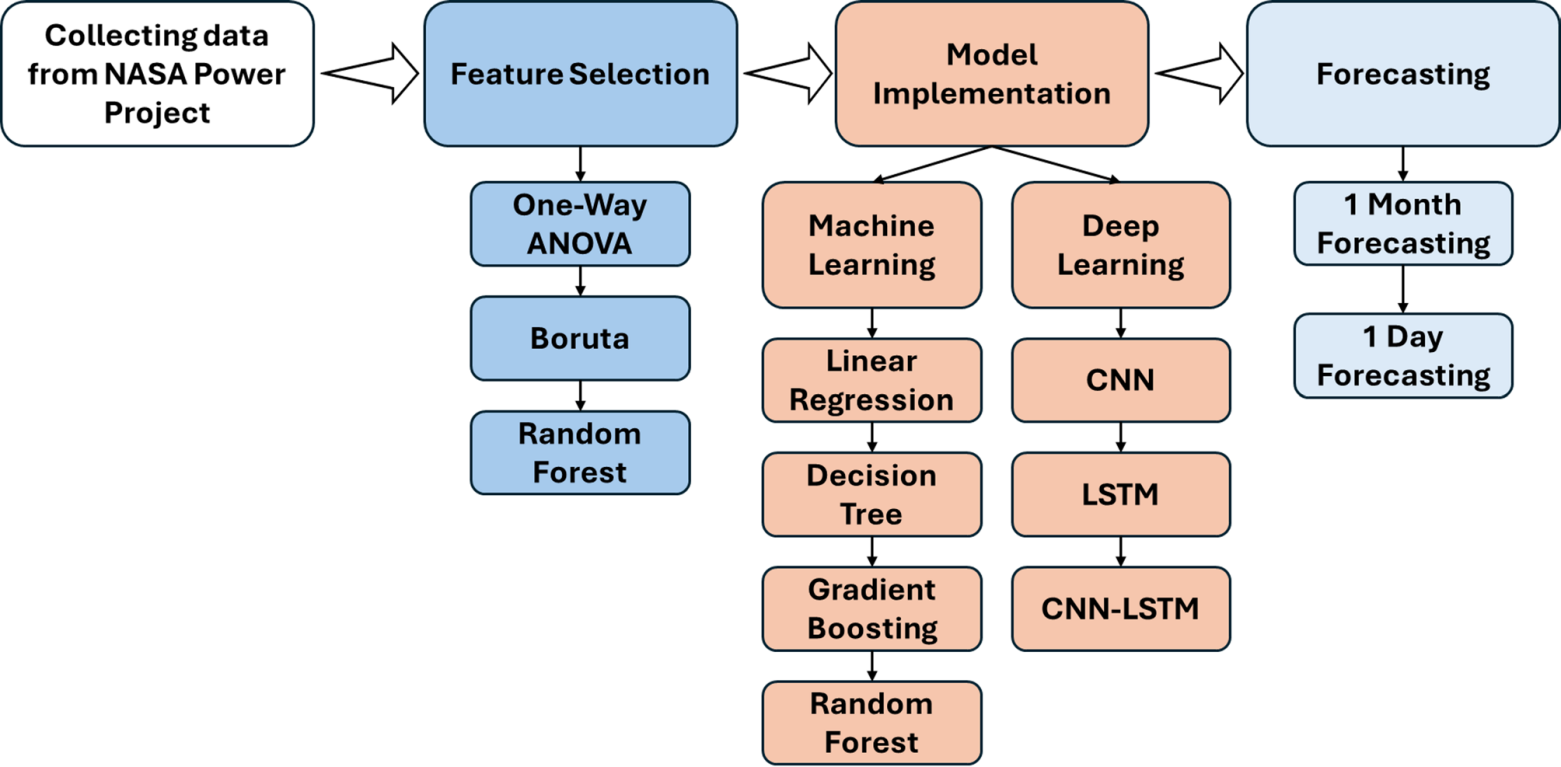
Schematic diagram for the workflow.

In this paper, the proposed approach consists of four main stages, as shown in Fig. 1. The stages begin with collecting data from the NASA Power project, then apply it with feature selection. The feature selection is implemented using one-way ANOVA, Boruta, and random forest. This stage is followed by utilizing machine learning and deep learning models for performance comparison and choosing the best model for enhancing the predictive accuracy of the system. The deep learning branch implements convolutional neural networks (CNN), long short-term memory (LSTM) networks, and a hybrid CNN-LSTM model to capture complex temporal and spatial dependencies within the data. In contrast, the machine learning branch employs Linear Regression, Decision Trees, Gradient Boosting, and Random Forest models, focusing on traditional regression approaches. Finally, the forecasting step is performed at two levels: one-day and one-month horizons, providing both short-term and long-term insights. Each step of the proposed approach is illustrated in detail in the following subsections.

### Dataset overview

The datasets used in this study are sourced from the NASA Power Project^28^, covering various timeframes: four years (2020–2023), three years (2021–2023), two years (2022–2023), and one year (2023). The four-year dataset consists of 35k records of hourly solar irradiance; the smaller datasets were derived as subsets from the four-year dataset. The dataset contained no missing data to handle; preprocessing was only applied to rename the features. Data collection was conducted at a specific location in Zafarana, Egypt, with coordinates 29°12’00.0"N 32°36’00.0"E. The region exemplifies the high solar potential found in Egypt’s Red Sea coastal regions. Its semi-arid climate, minimal cloud cover, and strategic location have already made it home to significant wind farm installations, with growing potential for solar energy development. These distinctive geographic and climatic characteristics make Zafarana an ideal case study for developing precise solar irradiance prediction models that could be applicable to similar high-potential solar regions globally. These datasets include hourly measurements of solar irradiance and related meteorological parameters, which are essentials for accurate solar energy predictions.

Nine features were selected from the NASA Power Datasets in Table 2 for their significance in predicting solar irradiance. These features capture key meteorological and temporal factors that impact solar energy production.

**Table 2:** Description of the used features.

Feature	Description
Year	The year in which the data was recorded.
Month	The month of the year, ranging from January to December.
Day	The day of the month, ranging from 1 to 31.
Hour	The time of the day, ranging from 0 to 23.
Temperature at 2 m	The air temperature was measured at a height of 2 m above the ground level.
Relative humidity at 2 m	The ratio of the current amount of moisture to the maximum amount it can hold at that temperature.
Surface pressure	The atmospheric pressure on the Earth’s surface.
Wind speeds at 10 m	The speed of the wind was measured at a height of 10 m above the ground.
Wind Direction at 10 m	The direction from which the wind is blowing at a height of 10 m above the ground.

### Feature selection methods

Feature selection is an essential step to monitor and select the features of importance to the target required. This step is critical for improving the system’s performance and reducing computational costs. In this study, three feature selection methods have been examined for different time periods (four years, three years, two years, one year): One-Way ANOVA, Boruta and Random Forest feature importance.

#### One-Way ANOVA

Represents a filter-based paradigm, selected for its simplicity and statistical use in evaluating linear relationships between features. It uses statistical methods to evaluate the significance of each feature by comparing the variance within each feature group to the variance between groups. It ranks features based on their contribution to the target variable^29^. The implementation utilized a function called SelectKBest with scoring to evaluate all nine features against the target variable. The features are then ranked according to their F-score, providing statistical measurements of the linear relationship between each feature and the target variable. It provides a baseline understanding of linear feature importance but misses interactions.

#### Boruta

A wrapper-based paradigm, chosen because it compares feature importance to randomized features, providing a robust way to handle non-linear relationships and interactions. It works as a wrapper around Random Forest. This implementation created shadow features, which is a randomized copy of the original features, and iteratively compared the importance of original features against these shadows over 100 iterations. Only features consistently outperforming their randomized counterparts were selected, with a ranking of 1 indicating significant features. This method offered a robust way to identify truly relevant predictors by accounting for feature interactions^29^. This method validates and refines One-Way ANOVA’s results by accounting for non-linear relationships via Random Forest’s ensemble approach.

#### Random forest

An embedded method intrinsic to tree-based models, selected for capturing non-linear dependencies and interactions automatically during model training. Feature importance was extracted directly from a trained Random Forest model, which measures importance based on the decrease in node impurity across all trees in the forest. This implementation provided insights into which features contribute most to prediction accuracy within the tree-based models. This approach offers a model-specific perspective, ensuring selected features align with the final predictive model.

### Model implementation

The methodology of this study consists of learning from historical data selected from the NASA Power project. Several machine learning and deep learning models were developed to predict solar irradiance effectively and are illustrated in the following subsections.

### Machine learning (ML) models

Various ML models are utilized in this stage based on effectiveness as concluded from the literature review, such as Linear Regression, Gradient Boosting, Decision Trees, and Random Forest. *Linear Regression* is a fundamental supervised learning algorithm designed for predicting continuous outcomes by modeling the relationship between feature variables and target variables. It achieves this by fitting a linear equation to the data, where the coefficients indicate the influence of each feature on the target variable. Linear regression performs well when relationships between variables are linear and straightforward. It is often a good choice for regression tasks due to its simplicity and interpretability^2^.

*Gradient boosting* is an ensemble technique that builds models sequentially. Each new model in the sequence aims to reduce the errors made by previous models, iteratively minimizing the loss function and enhancing prediction accuracy. Gradient boosting is highly effective at capturing complex patterns in data. It is well-suited for scenarios where high accuracy is critical^30^.

*Decision trees* provide a hierarchical approach used in the prediction. The model creates a tree-like structure where each node represents a feature, and each branch corresponds to a decision based on feature values. The final leaf node delivers the prediction. Decision trees handle non-linear data effectively and are easy to interpret. They are ideal for initial analysis and cases where interpretability is key, though they often benefit from pruning to improve generalization^31^.

*Random Forest* is an ensemble model that combines multiple decision trees to improve prediction robustness. Each tree in the forest is trained on a random subset of the data, with predictions averaged across all trees for final result. This approach enhances generalization by reducing variance, which mitigates the risk of overfitting. Random Forest is especially effective for high-dimensional data and when data relationships are non-linear. Random Forest tends to deliver strong, reliable performance across diverse applications, from classification to regression tasks^30^.

### Deep learning (DL) models

**Fig. 2:**
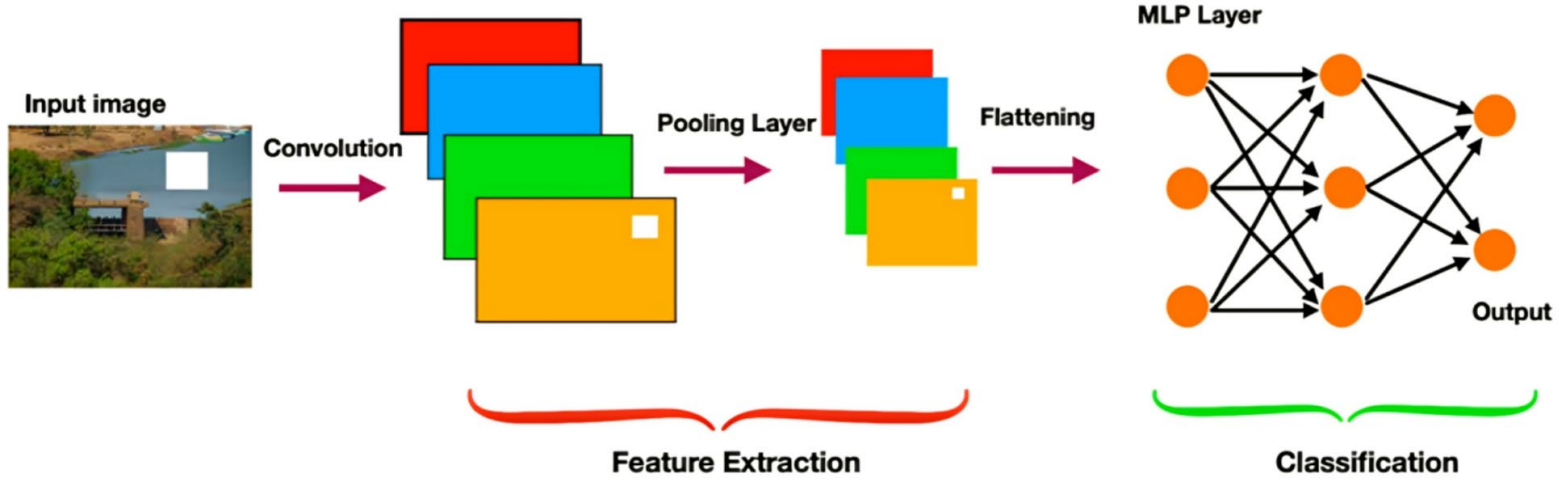
Foundation of CNN Architecture^32^.

In addition to the ML models, various DL models are implemented such as CNN, LSTM, and hybrid CNN-LSTM model. *CNN*, foundation in Fig. 2, is particularly well-suited for sequential data, making it effective in tasks like time series analysis and signal processing. By applying convolutional layers, the model automatically learns hierarchical feature representations from raw input data, which reduces the need for manual feature extraction. Pooling layers are added after each convolutional layer to reduce dimensionality, lower computational costs, and mitigating the risk of overfitting^3^. In this study, a 1D-CNN architecture with 64 neurons in a single convolutional layer is used, followed by a flatten layer and a fully connected layer for final predictions.

**Fig. 3:**
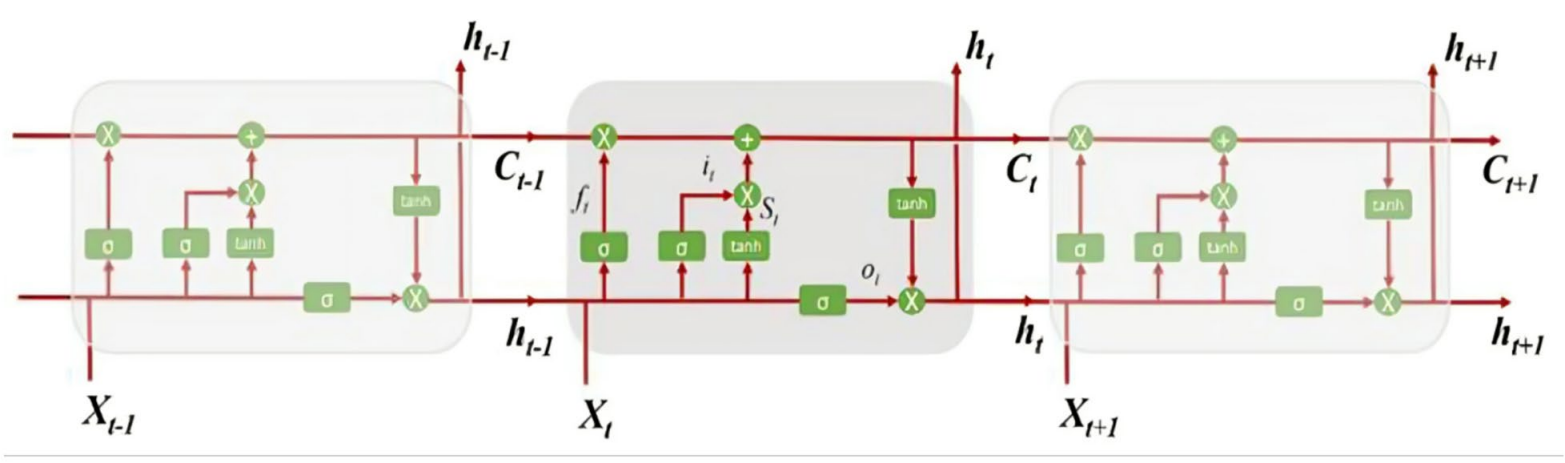
Architecture of LSTM^32^.

The *LSTM*, architecture in Fig. 3, is a type of recurrent neural network (RNN) optimized for learning dependencies over long sequences by using gating mechanisms—input, forget, and output gates—that allow the model to selectively retain or discard information as it processes each sequence. This functionality is especially beneficial for data with long-term dependencies, where important features can be scattered over time^33^. In our architecture, a single LSTM layer with 32 units followed by a fully connected layer is employed. This setup is designed to capture temporal dependencies effectively while avoiding excessive complexity, ensuring that the model focuses on patterns crucial for accurate prediction.

**Fig. 4:**
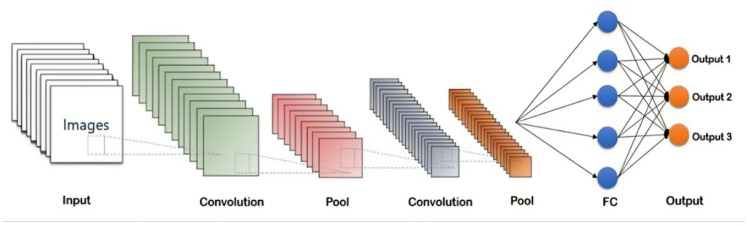
Structure of CNN-LSTM^32^.

*CNN-LSTM*, Structure shown in Fig. 4, is a hybrid model that combines the strengths of CNNs and LSTMs, integrating the feature extraction capabilities of CNNs with the temporal sequence modeling capabilities of LSTMs^29^. In the hybrid architecture, the initial CNN layer (with 64 neurons) captures spatial patterns in the sequential data, while the LSTM layer (32 units) that follows captures temporal dependencies. The CNN-LSTM model is particularly valuable when data has both spatial and temporal characteristics. The CNN-LSTM architecture provides a more comprehensive approach to modeling complex dependencies in the data.

### Models hyperparameter

To ensure reproducibility and transparency in comparative analysis, the hyperparameters and training configurations for all implemented models have been documented in Table 3. The machine learning models (Linear Regression, Gradient Boosting, Decision Tree, Random Forest) utilized scikit-learn’s default parameters unless specified otherwise, while deep learning architectures (CNN, LSTM, CNN-LSTM) shared consistent training protocols with early stopping to prevent overfitting.

**Table 3:** Hyperparameters and training configurations.

Model	Hyperparameters	Training Configuration
**Linear Regression**	fit_intercept = True copy_X = True	Fit directly on training data
**Gradient Boosting**	n_estimators = 100 learning_rate = 0.1 max_depth = 3	Fit on training data with default scikit-learn settings
**Decision Tree**	max_depth = None min_samples_split = 2	Fit on training data with default scikit-learn settings
**Random Forest**	n_estimators = 100 max_depth = None	Fit on training data with default scikit-learn settings
**1D-CNN**	1 Conv1D layer (64 filters, kernel_size = 3, ReLU) Flatten + Dense (1) output	Optimizer: Adam Loss: MSE EarlyStopping (patience = 2) Batch size: 32 Epochs: 1000 (early stopped)
**LSTM**	1 LSTM layer (32 units, ReLU) Dense (1) output	Optimizer: Adam Loss: MSE EarlyStopping (patience = 2) Batch size: 32 Epochs: 1000 (early stopped)
**CNN-LSTM**	1 Conv1D layer (64 filters, kernel_size = 3, ReLU) TimeDistributed(Flatten) LSTM (32, ReLU) Dense (1) output	Optimizer: Adam Loss: MSE EarlyStopping (patience = 2) Batch size: 32 Epochs: 1000 (early stopped)

### Key performance parameters

To evaluate the performance of the algorithms, four evaluation metrics commonly used like R² (R-Squared), MSE (Mean Square Error), RMSE (Root Mean Square Error), and MAE (Mean Absolute Error). To evaluate the accuracy of the models, R² metric was used. This regression analysis statistics measure indicates the proportion of variance in the dependent variable that is explained by the independent variable. As shown in Eq. (1), Where $\:S{S}_{res}$is the residual sum of squares, and $\:S{S}_{tot}$is the total sum of squares. The metrics calculating formula are shown as follows:

1 $$\:{R}^{2}\:\:=\:1-\:\frac{S{S}_{res}}{S{S}_{tot}}$$

2 $$MSE=\:\frac{1}{n}\sum\:_{i=1}^{n}{({y}_{i}-\widehat{{y}_{i}})}^{2}$$

3 $$RMSE=\:\sqrt{\frac{1}{n}\sum\:_{i=1}^{n}{({y}_{i}-\widehat{{y}_{i}})}^{2}}$$

4 $$MAE=\:\frac{1}{n}\sum\:_{i=1}^{n}|{y}_{i}-\widehat{{y}_{i}}|$$

Where $\:{y}_{i}$is the actual value and $\:{\widehat{y}}_{i}$is the predicted value and n is the number of data points. Equation (2) measures MSE as the average of squared errors. Equation (3) measures the RMSE that is the root average of the squared errors. Equation (4) calculates the average of absolute errors.

## Results and discussions

The simulations were conducted on an AMD Ryzen 5 4600 H with 16 GB RAMs and Nvidia GeForce GTX 1660 Ti GPU, and for software programs Python and TensorFlow framework were used with libraries like NumPy, Pandas, matplotlib.

### Feature selection

**Fig. 5:**
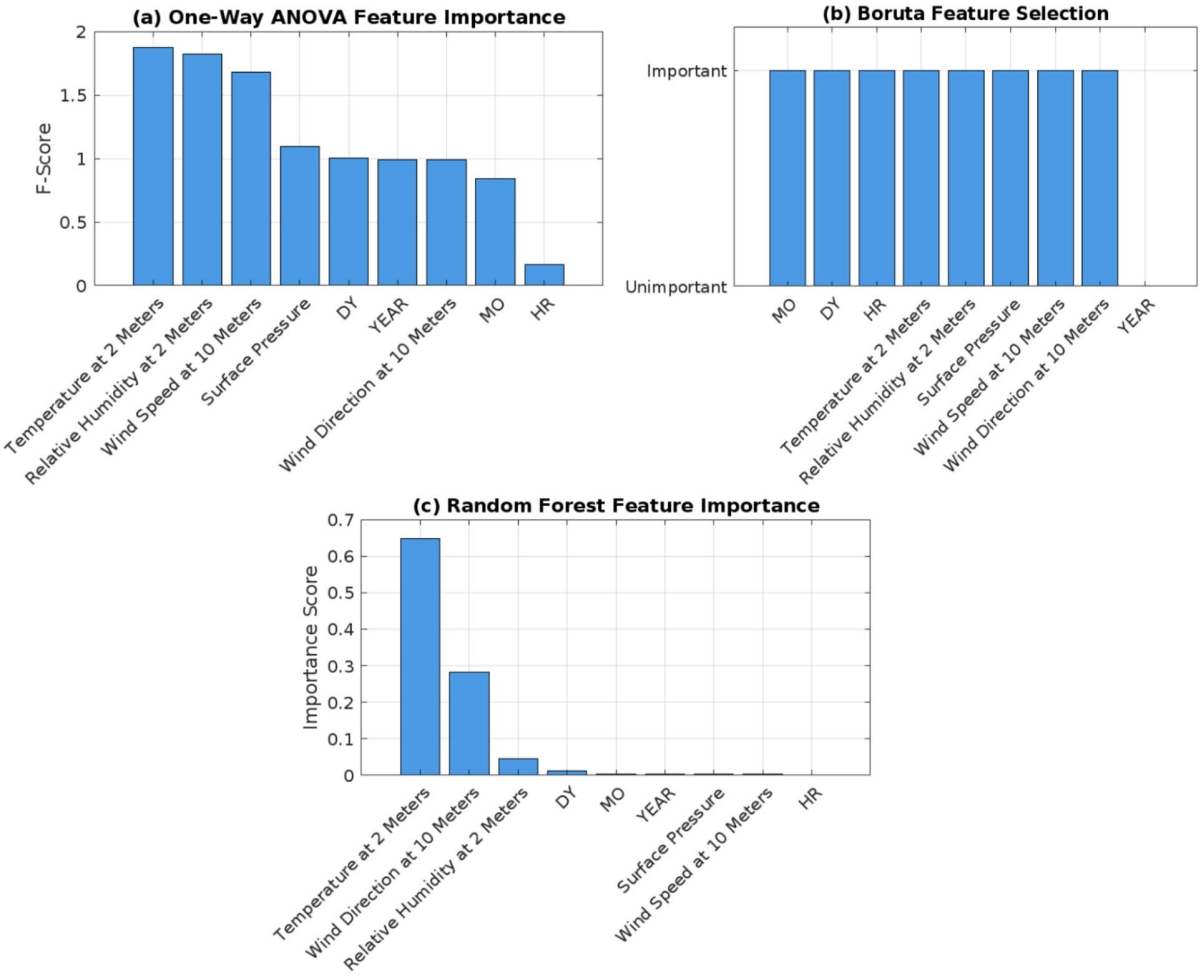
Feature selection methods in four years: (**a**) One-Way ANOVA Graph in Four Years, (**b**) Boruta Graph in Four Years, and. (**c**) Random Forest Graph in Four Years.

The primary analysis was conducted on the four-year dataset, the feature importance rankings were similar across other time periods, thus, four-year data is only shown. Figure 5 presents the importance of rankings for the four-year dataset across the three methods. The results from feature selection on the three-year, two-year, and one-year datasets largely align with those of the four-year dataset, showing consistent feature importance across methods. Minor differences for three-year and two-year dataset with similar feature rankings to the four-year dataset, with no significant deviations. One-year dataset (2023) has slightly different feature rankings, but temperature and humidity remain consistently high across methods.

One-way ANOVA pointed out that temperature, humidity, and wind speed as the most critical features for predicting solar irradiance, with hour ranked as the least important. Random forest ranks hour as the most significant feature, differing from One-Way ANOVA. Boruta marks the year feature as unimportant while highlighting key features without specific ranking.

### Model implementation and forecasting

The machine learning and deep learning models have been developed and trained on the 4 datasets mentioned in the earlier sections. For precise real-time forecasting the models were predicted for the next month (January 2024) in Table 4. Each model was evaluated across four metrics: R², MSE (Wh^2^/m^4^), RMSE (Wh/m^2^), MAE (Wh/m^2^), as shown in Table 4.

**Table 4:** Comparison for 2024’s first month Predictions.

		Machine Learning				Deep Learning		
Years	Metrics	Linear Regression	Gradient Boosting	Decision Tree	Random Forest	CNN	LSTM	CNN-LSTM
4	R2	58.4%	95.4%	96.3%	**97.8%**	42.9%	49.1%	51.7%
	MSE	23628.42	2562.1	2139.4	**1205.63**	32,445	28,911	27,417
	RMSE	153.71	50.61	46.25	**34.72**	180.12	170.03	165.58
	MAE	124.05	33.92	20.42	**15.77**	180.12	124.44	111.7
3	R2	55.5%	95.5%	95%	**97.8%**	50.8%	38.3%	21.2%
	MSE	25276.68	2537.5	2482.08	**1318**	34,083	35,798	28,693
	RMSE	158.98	50.37	49.82	**36.3**	184.61	189.2	169.39
	MAE	129.21	33.53	22.1	**16.26**	132.12	116.96	112.2
2	R2	59.6%	94.7%	96%	**97.7%**	45.8%	39.2%	50.2%
	MSE	22,927	2973.22	2283.46	**1287.6**	28,492	34,568	37,100
	RMSE	151.41	54.52	47.78	**35.88**	168.79	185.92	192.61
	MAE	121.84	37.17	22.62	**17.05**	123.84	110.5	129.65
1	R2	45.9%	54.5%	53.8%	**58.8%**	46.2%	25.4%	37.9%
	MSE	30,760	25,850	27,471	**23,201**	32,368	42,388	44,821
	RMSE	175.38	160.78	165.74	**152.3**	179.91	205.88	211.71
	MAE	144.08	105.59	104.06	**94.6**	140.55	138.02	131.55

As concluded from Table 4, Random Forest achieved high scores across metrics, with an $\:{R}^{2}$ exceeding 97% in three out of four periods. Decision Tree and Gradient Boosting also performed well, often close behind Random Forest in most metrics. Deep learning models displayed low scores, which might be due to the sensitivity of these models to temporal patterns and complex nonlinear relationships. The relative size of the dataset may limit the ability of deep learning models to learn temporal dependencies, as they often require orders of magnitude more data than traditional machine learning methods to achieve comparable performance. While LSTMs are theoretically capable of learning long-term dependencies, their performance is highly sensitive to hyperparameter. Our LSTM (32 units) may not have sufficient capacity to model both short-term and long-term trends simultaneously.

To evaluate the improvement of Random Forest over other models, paired t-tests were conducted on the 4-year dataset. The tests compared Random Forest against Linear Regression, Gradient Boosting, Decision Tree, and deep learning models. Results (Table 5) show that Random Forest performed similarly to Linear Regression (*p* = 0.969), Gradient Boosting (*p* = 0.997), and Decision Tree (*p* = 0.571). Surprisingly, Random Forest errors were significantly larger than LSTM (*p* < 0.0001, t = − 9.57) and CNN-LSTM (*p* < 0.0001, t = − 12.19), contradicting R² metrics in Table 4. This discrepancy suggests deep learning models better capture nonlinear patterns in raw errors.

**Table 5:** Paired t-test results (Random forest vs. other models).

Comparison	t-statistic	*p*-value	Conclusion
RF vs. LR	0.0385	0.9693	No significant difference
RF vs. GB	−0.0042	0.9966	No significant difference
RF vs. DT	0.5662	0.5713	No significant difference
RF vs. CNN	3.9500	0.0001	RF errors are smaller than CNN
RF vs. LSTM	−9.5723	< 0.0001	RF errors are larger than LSTM
RF vs. CNN-LSTM	−12.1916	< 0.0001	RF errors are larger than CNN-LSTM

While RF achieved the highest R² (Table 4), t-tests revealed that LSTM and CNN-LSTM produced significantly smaller prediction errors. This suggests RF better explains variance in irradiance trends, whereas deep learning models minimize absolute deviations. The choice between RF and deep learning may depend on the application: RF for overall trend accuracy, or LSTM/CNN-LSTM for pointwise error reduction.

### Validation of feature selection methods

Due to the difference between feature importance methods, it’s crucial to involve testing the model’s performance by excluding each feature individually to assess its impact directly. The feature selection methods provided an overall ranking based on their algorithms. However, the importance of features may vary depending on the method used.

**Fig. 6:**
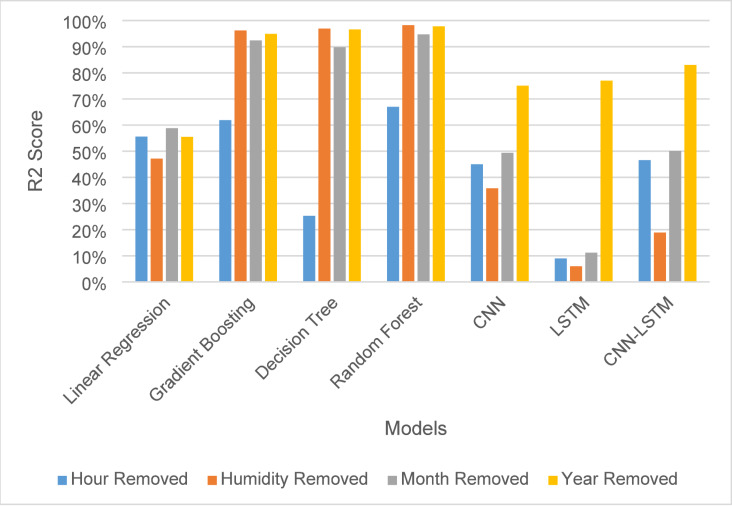
R2 score for solar irradiance forecasting for a month after removing features.

The analysis identified the three most important features and one least important in the 4 years period, and each was removed individually to assess its impact. Removing the hour feature, as shown in Fig. 6; Table 6, led to a significant decrease in $\:{R}^{2}$ score across all models, indicating its high importance, except for linear regression and CNN, where the decrease was minimal.

**Table 6:** Comparison for 2024’s first month predictions after removing hour feature.

Years	Metrics	Linear Regression	Gradient Boosting	Decision Tree	Random Forest	CNN	LSTM	CNN-LSTM
4	R2	55.6%	61.9%	25.3%	**67%**	45%	9%	46.6%
	MSE	25,208	21,667	42,479	**18,657**	31,276	51,645	30,363
	RMSE	158.77	147.19	206.1	**136.59**	176.85	227.25	174.25
	MAE	130.1	109.16	117.18	**92.73**	129.94	207.49	120.8
3	R2	52.4%	61.6%	14.4%	**65.5%**	41.6%	48.2%	−13%
	MSE	27,021	21,805	48,686	**19,598**	33,181	29,442	64,403
	RMSE	164.38	147.66	220.64	**139.99**	182.15	171.58	253.77
	MAE	135.32	110.47	131.69	**95.8**	134.17	134.56	239.1
2	R2	57.3%	59.9%	17%	**65.2%**	49.4%	43.8%	37.8%
	MSE	24,261	22,762	47,184	**19,751**	28,748	31,909	35,345
	RMSE	155.75	150.87	217.21	**140.54**	169.55	178.63	188
	MAE	126.68	109.91	129.54	**95.72**	131.98	128.69	124.82
1	R2	44.1%	61%	26%	**62.7%**	41.1%	26.1%	45.9%
	MSE	31,766	22,130	42,049	**21,167**	33,459	41,984	30,771
	RMSE	178.23	148.76	205.05	**145.48**	182.92	204.9	175.41
	MAE	147.19	110.35	121.11	**102.01**	150.89	182.6	133.99

Removing the humidity and month features, in Fig. 6, caused only minor changes, with the month feature leading to humidity making it particularly relevant to tree-based models such as Gradient Boosting, Decision Tree, and Random Forest. The year feature had no noticeable effect on the machine learning models but substantially improved the performance of deep learning models, highlighting its influence when included in the data, as shown in Table 7.

**Table 7:** Comparison for 2024’s first month predictions after removing year Feature.

Year	Metrics	Linear Regression	Gradient Boosting	Decision Tree	Random Forest	CNN	LSTM	CNN-LSTM
4	R2	55.5%	94.9%	96.6%	**97.8%**	75.1%	77%	83%
	MSE	25,279	2882	1965	**1149**	14,160	12,970	9461
	RMSE	158.99	53.68	44.33	**33.9**	118.99	113.88	97.26
	MAE	128.81	36.85	19.92	**14.99**	97.25	82.25	57.86
3	R2	57.2%	95.8%	95.2%	**97.7%**	76.5%	84.5%	72.5%
	MSE	24,330	2376	2723	**1305**	13,355	8764	15,637
	RMSE	155.98	48.74	52.18	**36.13**	115.56	93.62	125.04
	MAE	126.49	31.5	22.11	**15.82**	94.05	63.44	84.14
2	R2	52.1%	94.8%	96.1%	**97.7%**	73.4%	84.3%	79.6%
	MSE	27,193	2955	2188	**1300**	15,084	8875	11,553
	RMSE	164.9	54.36	46.77	**36.05**	122.82	94.2	107.48
	MAE	133.96	37.06	21.85	**17.05**	100.7	60.73	66.63
1	R2	48%	54.5%	55.1%	**58.1%**	41.9%	66.8%	77.3%
	MSE	29553.12	25850.79	25523.93	**23819.82**	32,991	18,864	12,904
	RMSE	171.91	160.78	159.76	**154.33**	181.63	137.34	113.59
	MAE	141.06	105.59	100.49	**96.25**	129.53	94.8	67.88

**Fig. 7:**
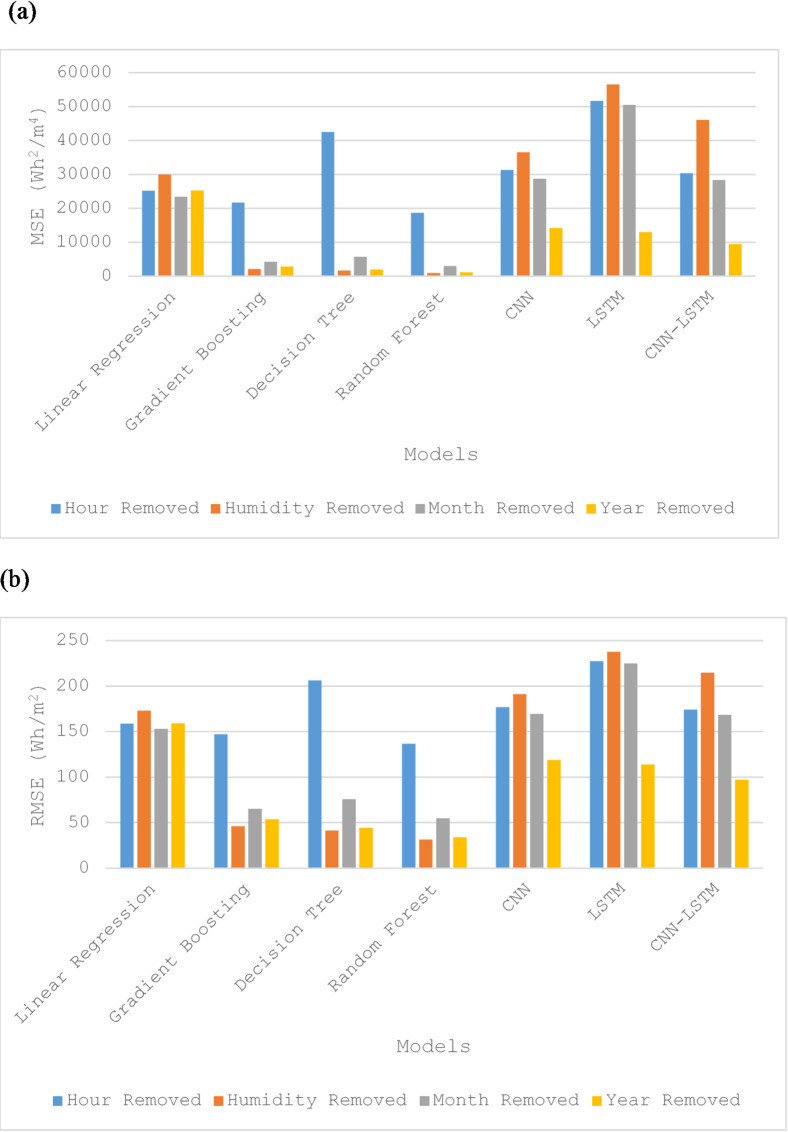
Forecasting for a Month After Removing Features Respectively:. (**a**) MSE for Solar Irradiance Forecast, (**b**) RMSE for Solar Irradiance Forecast.

In terms of MSE and RMSE, removing the hour feature had a small impact on most machine learning algorithms except for Decision Tree, where it caused a notable effect, and a significant impact on deep learning models, as shown in Fig. 7-a and -b. Removing humidity and month led to a significant improvement in tree-based algorithms but caused major declines in performance for deep learning models, with only minor changes in linear regression. Removing the year features improved slightly for all models, especially deep learning models except linear regression.

**Fig. 8:**
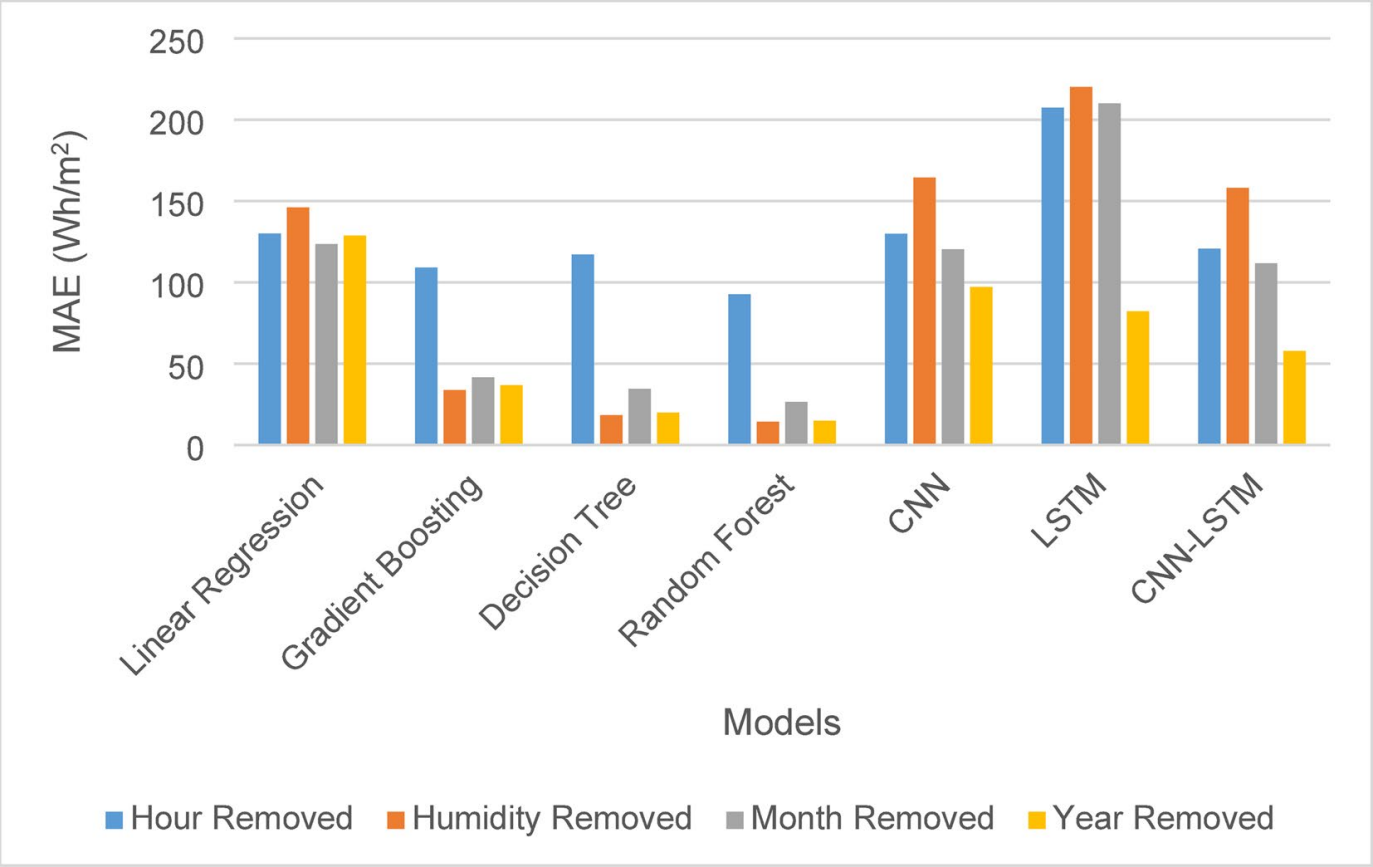
MAE for solar irradiance forecasting for a month forecasting after removing features.

In terms of MAE, the removal of the hour feature had a substantial impact, underscoring its significance within the dataset, as shown in Fig. 8. Removing humidity and month had minimal impact on machine learning models but a notable effect on deep learning models. Additionally, the exclusion of the year feature led to a noticeable change in MAE, as illustrated in Fig. 8.

Based on the results and analysis discussed after removing each feature individually, it appears that Random Forest feature selection is the optimal method used. However, in terms of $\:{R}^{2}$ the removal of month features affected the data by decreasing in performance unlike relative humidity didn’t affect the data properly, which means that from the analysis obtained, the month feature is second most important feature in the dataset instead of the humidity feature as taking from the ranking of Random Forest feature importance.

**Fig. 9:**
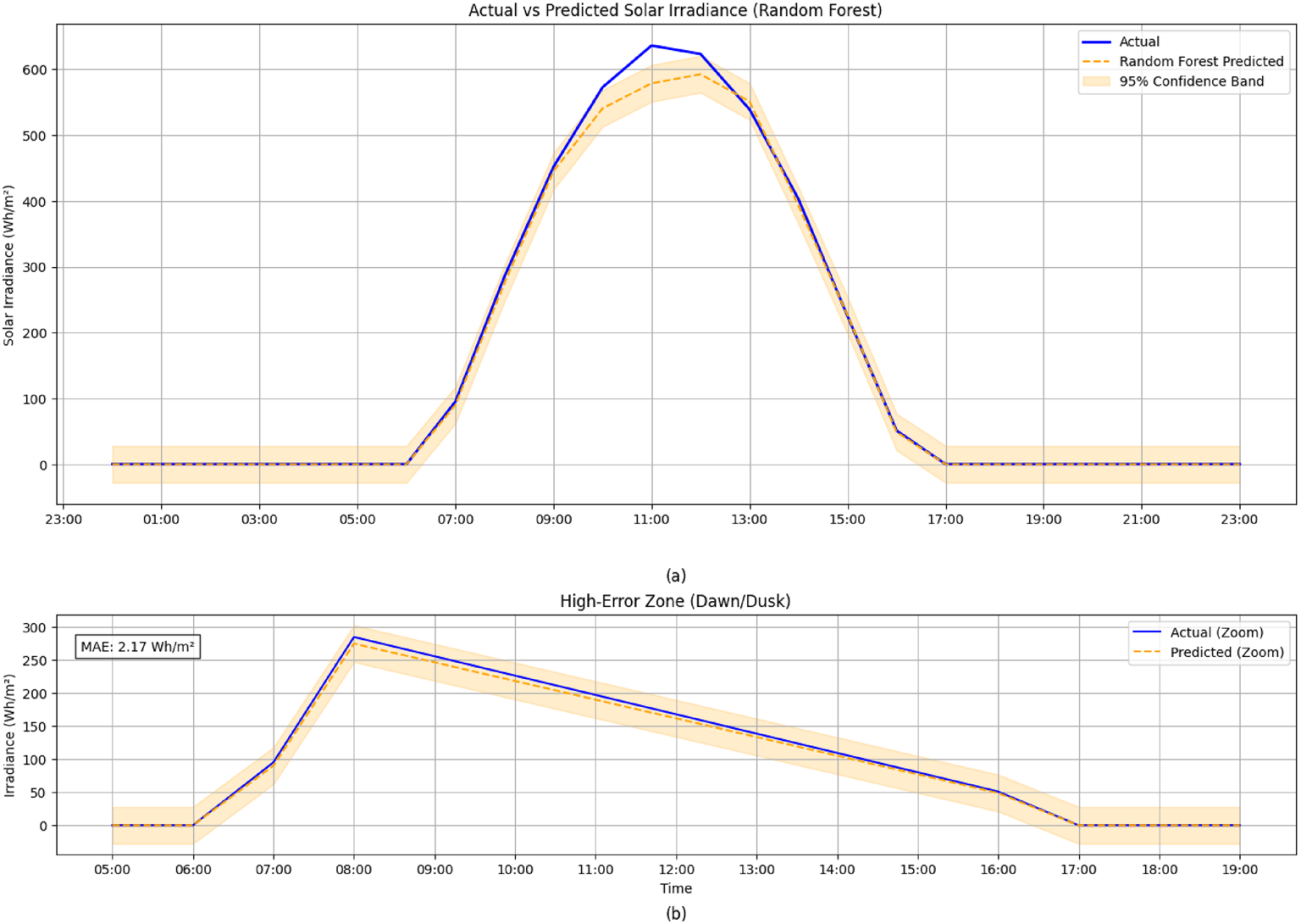
Actual vs. Predicted Solar Irradiance Using Random Forest for (**a**) Full Period One Day (**b**) High-Error Zones.

The model implementation stage is followed by analyzing and forecasting the solar irradiation for a whole month and a day by feeding the data to the machine learning and deep learning models and comparing the forecast performance evaluation with the actual driven data, as shown in Fig. 9-a and -b.

## Conclusion

This paper evaluated a diverse set of machine learning and deep learning models for forecasting solar irradiance in Zafarana, Egypt. Leveraging historical meteorological data from the NASA Power Project, various feature selection techniques are applied, including One-Way ANOVA, Boruta, and Random Forest, to identify the most influential features affecting solar irradiance, which contributed to the overall performance of the models. Random Forest feature importance identified temporal (hour, month) and meteorological (temperature, humidity) variables as most influential. Our findings indicate that traditional machine learning models, particularly Random Forest and Gradient Boosting, delivered high predictive accuracy. However, deep learning architectures, such as LSTM and the CNN-LSTM hybrid, did not achieve the expected improvements, suggesting their complexity may require richer datasets or hyperparameter tuning for this geographic context. Machine learning models achieved the highest predictions in both short-term (one-day-ahead) and long-term (one-month-ahead) periods with R2 score of 0.9948 and 0.978, respectively, providing robust insights into solar irradiance across different timeframes. Statistical tests confirmed that Random Forest’s performance is comparable to tree-based models (Gradient Boosting, Decision Tree) but inferior to deep learning (LSTM, CNN-LSTM) in minimizing prediction errors. However, RF remains advantageous for interpretability and computational efficiency, particularly when variance explanation (R²) is prioritized over pointwise accuracy. This research contributes to the growing field of solar energy forecasting by demonstrating the efficacy of feature selection combined with machine learning techniques, which may offer reliable and efficient alternatives to more computationally intensive deep learning models for similar environmental applications.

## Data Availability

The dataset that supports the finding of this study is being available online on NASA POWER project data viewer “https://power.larc.nasa.gov/”.

## References

[1] Zhang, Q., Yan, H. & Liu, Y. Power generation forecasting for solar plants based on dynamic bayesian networks by fusing multi-source information. *Renew. Sustain. Energy Rev.* **202**, 114691 (2024).

[2] Jebli, I., Belouadha, F. Z., Kabbaj, M. I. & Tilioua, A. Prediction of solar energy guided by pearson correlation using machine learning. *Energy* **224**, 120109 (2021).

[3] Lara-Benítez, P., Carranza-García, M., Luna-Romera, J. M. & Riquelme, J. C. Short-term solar irradiance forecasting in streaming with deep learning. *Neurocomputing* **546**, 126312 (2023).

[4] Ribeiro, R. & Fanzeres, B. Identifying representative days of solar irradiance and wind speed in Brazil using machine learning techniques. *Energy AI* **15**, 100320 (2024).

[5] Hanif, M. F. et al. Advancing solar energy forecasting with modified ANN and light GBM learning algorithms. *AIMS Energy*. **12**, 350–386 (2024).

[6] Hari, N. G. & Jisha, G. Solar Irradiance Prediction using Deep Learning-Based Approaches. in *Proceedings of IEEE Asia-Pacific Conference on Computer Science and Data Engineering, CSDE 2022*Institute of Electrical and Electronics Engineers Inc., (2022). 10.1109/CSDE56538.2022.10089282

[7] Nkounga, W. M. et al. Short-Term Multi Horizons Forecasting of Solar Irradiation Based on Artificial Neural Network with Meteorological Data: Application in the North-west of Senegal. in. *16th International Conference on Ecological Vehicles and Renewable Energies, EVER 2021* (Institute of Electrical and Electronics Engineers Inc., 2021). (2021). 10.1109/EVER52347.2021.9456600

[8] Soni, S. & Bindal, R. K. Selection of Area and Collect Required Data for Power Prediction of a Solar Plant. in *2nd Edition of IEEE Delhi Section Owned Conference, DELCON 2023 - Proceedings*Institute of Electrical and Electronics Engineers Inc., (2023). 10.1109/DELCON57910.2023.10127372

[9] Fatima-ezzahra, D., Abdellah, B. & Abdellatif, G. Estimation of ultraviolet solar irradiation of semi-arid area - case of Benguerir-. in *The 4th edition of The International Conference on Electrical and Information Technologies, ICEIT 20*IEEE, Morocco, (2020).

[10] Muhammad Farhan et al. Enhancing solar forecasting accuracy with sequential deep artificial neural network and hybrid random forest and gradient boosting models across varied terrains. *Adv. Theory Simul.* **7**, 2470015 (2024).

[11] Pardeep Singla, S., Saroha, M. & Duhan, V. S. Kalyan Singh. A solar irradiance forecasting model using iterative filtering and bidirectional long short-term memory. *Energy Sour. Part A Recover. Utilization Environ. Eff.* **46**, 8202–8222 (2024).

[12] Singla, P., Duhan, M. & Saroha, S. Solar irradiation forecasting by long-short term memory using different training algorithms. Renewable Energy Optimization, Planning and Control. Studies in Infrastructure and Control. Springer, Singapore. 10.1007/978-981-16-4663-8_7

[13] Singla, P., Duhan, M. & Saroha, S. A point and interval forecasting of solar irradiance using different decomposition based hybrid models. *Earth Sci. Inf.* **16**, 2223–2240 (2023).

[14] Bhatt, A., Ongsakul, W., Nimal Madhu, M. & Singh, J. G. Sliding window approach with first-order differencing for very short-term solar irradiance forecasting using deep learning models. *Sustain. Energy Technol. Assess.* **50**, 101864 (2022).

[15] Irshad, K. et al. Arithmetic optimization with hybrid deep learning algorithm based solar radiation prediction model. *Sustain. Energy Technol. Assess.* **57**, 103165 (2023).

[16] Zhao, X., Xie, L., Wei, H., Wang, H. & Zhang, K. Fuzzy inference systems based on multi-type features fusion for intra-hour solar irradiance forecasts. *Sustain. Energy Technol. Assess.* **45**, 101061 (2021).

[17] Yang, L., Gao, X., Hua, J. & Wang, L. Intra-day global horizontal irradiance forecast using FY-4A clear Sky index. *Sustain. Energy Technol. Assess.* **50**, 101816 (2022).

[18] Teyabeen, A. A., Elhatmi, N. B., Essnid, A. A. & Mohamed, F. Comparison of Seven Empirical Models For Estimating Monthly Global Solar Radiation, (Case Study: Libya). in *2021 12th International Renewable Energy Congress, IREC 2021*Institute of Electrical and Electronics Engineers Inc., (2021). 10.1109/IREC52758.2021.9624843

[19] Budiyanto, M. A. & Lubis, M. H. Comparison Result of Hourly Solar Radiation under the Clear Sky Condition Based on of Solar Radiation Model and Measured Data Experiment. in *Proceeding – 1st International Conference on Information Technology, Advanced Mechanical and Electrical Engineering, ICITAMEE 2020* 298–302Institute of Electrical and Electronics Engineers Inc., (2020). 10.1109/ICITAMEE50454.2020.9398403

[20] Nawab, F., Ibrahim, A., Zeeshan Suheel, S. & Ahmed Goje, A. Comparison of ANN Global Horizontal Irradiation predictions with Satellite Global Horizontal Irradiation using Statistical evaluation. in *4th International Conference on Computing, Mathematics and Engineering Technologies: Sustainable Technologies for Socio-Economic Development, iCoMET 2023* (Institute of Electrical and Electronics Engineers Inc., 2023). (Institute of Electrical and Electronics Engineers Inc., 2023). (2023). 10.1109/iCoMET57998.2023.10099300

[21] Rehiara, A. B. & Setiawidayat, S. Day Ahead Solar Irradiation Forecasting Based on Extreme Learning Machine. in *Proceedings – 2022 IEEE International Conference on Cybernetics and Computational Intelligence, CyberneticsCom 2022* 63–66Institute of Electrical and Electronics Engineers Inc., (2022). 10.1109/CyberneticsCom55287.2022.9865532

[22] Halima, D. et al. Solar radiation Estimation based on a new combined approach of artificial neural networks (ANN) and genetic algorithms (GA) in South Algeria. *Computers Mater. Continua*. **79**, 4725–4740 (2024).

[23] Hanif, M. F. & Mi, J. Harnessing AI for solar energy: Emergence of transformer models. *Appl. Energy* **369**, 123541 (2024).

[24] Naveed, M. S. et al. Leveraging advanced AI algorithms with transformer-infused recurrent neural networks to optimize solar irradiance forecasting. *Front. Energy Res.* **12**, 1485690. 10.3389/fenrg.2024.1485690 (2024).

[25] Lakhan, A., Mohammed, M. A., Kozlov, S. & Rodrigues, J. J. P. C. Mobile-fog-cloud assisted deep reinforcement learning and blockchain-enable IoMT system for healthcare workflows. *Trans. Emerg. Telecommun. Technol.* **35**, e4363 (2024).

[26] Lu, W., Zhao, H., He, Q., Huang, H. & Jin, X. Category-consistent deep network learning for accurate vehicle logo recognition. *Neurocomputing* **463**, 623–636 (2021).

[27] Kumar, A. et al. Improved CNN for the diagnosis of engine defects of 2-wheeler vehicle using wavelet synchro-squeezed transform (WSST). *Knowl. Based Syst.* **208**, 106453 (2020).

[28] NASA Power Data. https://power.larc.nasa.gov/

[29] Taha, A., Nazih, N. & Saad, A. Strategies for Assessing Pain using Multimodal Pain Detection. in *2024 International Telecommunications Conference (ITC-Egypt)*IEEE, (2024).

[30] Yagli, G. M., Yang, D. & Srinivasan, D. Automatic hourly solar forecasting using machine learning models. *Renew. Sustain. Energy Rev.* **105**, 487–498 (2019).

[31] Ramos, D., Faria, P., Morais, A. & Vale, Z. Using decision tree to select forecasting algorithms in distinct electricity consumption context of an office Building. *Energy Rep.* **8**, 417–422 (2022).

[32] Nadeem, A. et al. AI-Driven precision in solar forecasting: breakthroughs in machine learning and deep learning. *AIMS Geosci.* **10**, 684–734 (2024).

[33] Ntlela, S. A. & Davidson, I. E. Solar Irradiation Forecasting for the City of Durban Using Time Series Analysis. in *Proceedings – 30th Southern African Universities Power Engineering Conference, SAUPEC* (Institute of Electrical and Electronics Engineers Inc., 2022). (Institute of Electrical and Electronics Engineers Inc., 2022). (2022). 10.1109/SAUPEC55179.2022.9730711

